# Enhancement in surface mobility and quantum transport of Bi_2−x_Sb_x_Te_3−y_Se_y_ topological insulator by controlling the crystal growth conditions

**DOI:** 10.1038/s41598-018-35674-z

**Published:** 2018-11-23

**Authors:** Kyu-Bum Han, Su Kong Chong, Anton O. Oliynyk, Akira Nagaoka, Suzanne Petryk, Michael A. Scarpulla, Vikram V. Deshpande, Taylor D. Sparks

**Affiliations:** 1 Department of Materials Science and Engineering, University of Utah, Salt Lake City, Utah 84112 USA; 2 Department of Physics and Astronomy, University of Utah, Salt Lake City, Utah 84112 USA; 3 Department of Chemistry, University of Alberta, Edmonton, AB T6G 2G2 Canada; 4 Department of Electrical Engineering, University of Utah, Salt Lake City, Utah 84112 USA; 5 Department of Computer Science, Cornell University, 402 Gates Hall, Ithaca, NY 14853 USA

## Abstract

Despite numerous studies on three-dimensional topological insulators (3D TIs), the controlled growth of high quality (bulk-insulating and high mobility) TIs remains a challenging subject. This study investigates the role of growth methods on the synthesis of single crystal stoichiometric BiSbTeSe_2_ (BSTS). Three types of BSTS samples are prepared using three different methods, namely melting growth (MG), Bridgman growth (BG) and two-step melting-Bridgman growth (MBG). Our results show that the crystal quality of the BSTS depend strongly on the growth method. Crystal structure and composition analyses suggest a better homogeneity and highly-ordered crystal structure in BSTS grown by MBG method. This correlates well to sample electrical transport properties, where a substantial improvement in surface mobility is observed in MBG BSTS devices. The enhancement in crystal quality and mobility allow the observation of well-developed quantum Hall effect at low magnetic field.

## Introduction

Three-dimensional topological insulator (3D TI) is a novel state of quantum matter which shows potential applications in spintronics^1,2^ and quantum computing^3,4^ owing to the unique spin-momentum locked surface states. Charge transport in such Dirac dispersion surface states is less sensitive than ordinary conductive materials to defects because the surface states are protected by time-reversal symmetry^5^. The surfaces states in 3D TI have been extensively studied and confirmed using angle-resolved photoemission spectroscopy^6,7^, scanning tunneling microscopy^8^ and electric transport measurements^9,10^. However, the typical strong 3D TIs, Bi_2_Se_3_ and Bi_2_Te_3_ suffer from their deep bulk-doping^11^. The difficulty in isolating bulk contributions from surface state transport limits the implementation of TIs in electronic devices^12^. More recent studies focus on the ternary and quaternary tetradymite Bi-based TI, namely Bi_2_Te_2_Se (BTS)^13–17^ and Bi_2−*x*_Sb_*x*_Te_3−*y*_Se_*y*_^18–25^, which show a large bulk resistivity, ambipolar surface transport and clear quantum oscillations. Particularly, the stoichiometric BiSbTeSe_2_ (BSTS) is a very promising TI candidate as it manifests high quality integer quantum Hall effect originating from its surface states^23–25^.

**Figure 1:**
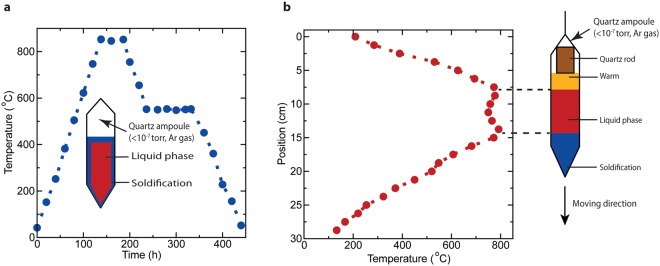
The measured temperature profiles of the melting (**a**) and vertical Bridgman (**b**) furnaces. The schematics of ampoules in (**a**,**b**) illustrate the crystal growth mechanism for the respective methods.

Various growth techniques have been reported to prepare BSTS single crystals. Among those, melting^17–22,24,25^ and vertical Bridgman^13,14,23^ methods are the most widely used synthetic methods. The single crystal growth methodology for melting method (as illustrated in Fig. 1(a)) generally involves the following procedures: (i) Melting and mixing the source materials in a sealed ampoule at a temperature above the melting temperature. (ii) The molten materials are annealed at a temperature just above the melting point for a long period to allow atomic diffusion and nucleation of a crystalline lattice. (iii) The materials are cooled down to the room temperature at a very slow rate to minimize additional nucleation sites in favor of growing the existing single crystal. Obviously, melting growth can cause polycrystalline ingot as there is no fixed single nucleation site. However, numerous literature has claimed that they achieved single crystal growth using the melting method^17–22,24,25^. On the other hand, the vertical Bridgman method applies a vertical motion to translate the materials along a temperature gradient from hot to cold zones at a very slow rate, as shown in Fig. 1(b). The source materials are gradually melted and solidified from the bottom to the top of an ampoule to yield single crystal growth. The crystal quality greatly depends on the translation rate as the slow solidification encourages the sample homogeneity and minimizes the crystal defects^26^.

Control of stoichiometry of the Bi_2-*x*_Sb_*x*_Te_3-*y*_Se_*y*_ is crucial to control the chemical potential of the surface states to be within the bulk gap^19^. Despite many studies, the single crystal growth of stoichiometric BSTS with remains challenging. Two processing conditions have not been fully explored in BSTS growth which may play an important role in crystal quality: (i) Rather than melting elemental precursors, it is possible to pre-react precursors to form a more homogenous compound prior to single crystal growth. (ii) Limiting the empty ampoule headspace. For the BSTS system, the starting materials have very different melting temperatures (T_m_) ranging from 220 °C (Se) to 631 °C (Sb). Therefore, an initial mixing step is crucial to yield highly stoichiometric and homogeneous BSTS single crystals. In the mixing process, the starting materials are melted and stirred multiple times over a period at high temperature of 850 °C to obtain the homogeneity^18–20^. For the second aspect (suitable only to vertical Bridgman growth), a quartz rod is inserted at the top of the BSTS polycrystalline ingot to reduce the space for evaporating materials, and thus minimize the loss of low T_m_ elements (refer to schematic in Fig. 1(b)). As the first and second aspects can only be applied separately to the melting and Bridgman growth methods, it is practical to investigate a two-step growth technique by combining melting and Bridgman methods.

In this work, we provide a systematic study on the crystal growth of BSTS using three different methods, namely melting growth (MG), vertical Bridgman growth (BG), and two-step melting-Bridgman growth (MBG). The MG and BG parameters are illustrated in Fig. 1(a,b), respectively. MBG begins with growing an ingot via MG (possibly single crystal), and then follows with recrystallization into a single crystal by BG. The single crystal BSTS samples grown by MG, BG and MBG methods are shown in Fig. S1. The composition homogeneity, crystal structure and low temperature magnetoelectric transport of the three types of BSTS samples are characterized. We show that the structural and electrical transport properties depend strongly on the growth method. The crystallinity of the BSTS, identified by structural properties, is correlated to the surface mobility and the quantum transport properties.

## Results

### Stoichiometry study

The elemental composition of the as-grown BSTS samples was investigated using both energy-dispersive X-ray spectroscopy (EDS) and inductively coupled plasma mass spectrometry (ICP-MS). The results are presented in Table 1. The EDS data were taken from the homogeneous part of the crystals (excluding the side surfaces). Typically, the crystal formed a cone shape with about 1 cm in diameter and about 1–2 cm in height. The single BSTS crystals shown in Fig. S1 are the pieces after cleaving the original samples. The region of EDS study is about 0.5 × 0.5 cm^2^, near the center of the crystal. The EDS results are presented in the ratio of Bi:Sb:Te:Se converted from the atomic percentage of the quantitative data. The BSTS crystals grown by both MG and BG methods showed similar composition ratios which differed from MBG method. The composition of the MBG sample was found to be ~10% closer to the stoichiometric ratio (1:1:1:2 for Bi:Sb:Te:Se) compared to the MG and BG samples. To further confirm the composition results, we utilized the more accurate ICP-MS for the elemental characterization. Consistent with the EDS analysis, both MG and BG samples showed similar composition in the ICP-MS spectrum and MBG sample was again found to more closely match the desired stoichiometric BiSbTeSe_2_. Although the MBG sample was grown from the MG crystal, which is showing slightly off stoichiometry in the center part of the crystal. We emphasize that the second growth step was done by using the whole MG crystal where we expect the off stoichiometry is balanced by sides area of the crystal. The second step Bridgman growth resulted in more stoichiometric and homogenous BSTS crystal.

**Table 1:** The elemental composition measured using EDS and ICP-MS for different methods prepared BSTS samples.

Growth method	EDS (±2%)	ICP-MS (±3%)
MG	Bi_1.00_Sb_0.90_Te_0.95_Se_2.15_	Bi_0.75_Sb_1.25_Te_0.56_Se_2.44_
BG	Bi_0.92_Sb_0.95_Te_0.98_Se_2.15_	Bi_0.79_Sb_1.21_Te_0.62_Se_2.38_
MBG	Bi_0.96_Sb_1.02_Te_0.97_Se_2.05_	Bi_0.89_Sb_1.11_Te_0.88_Se_2.12_

### Crystallinity evaluation

**Figure 2:**
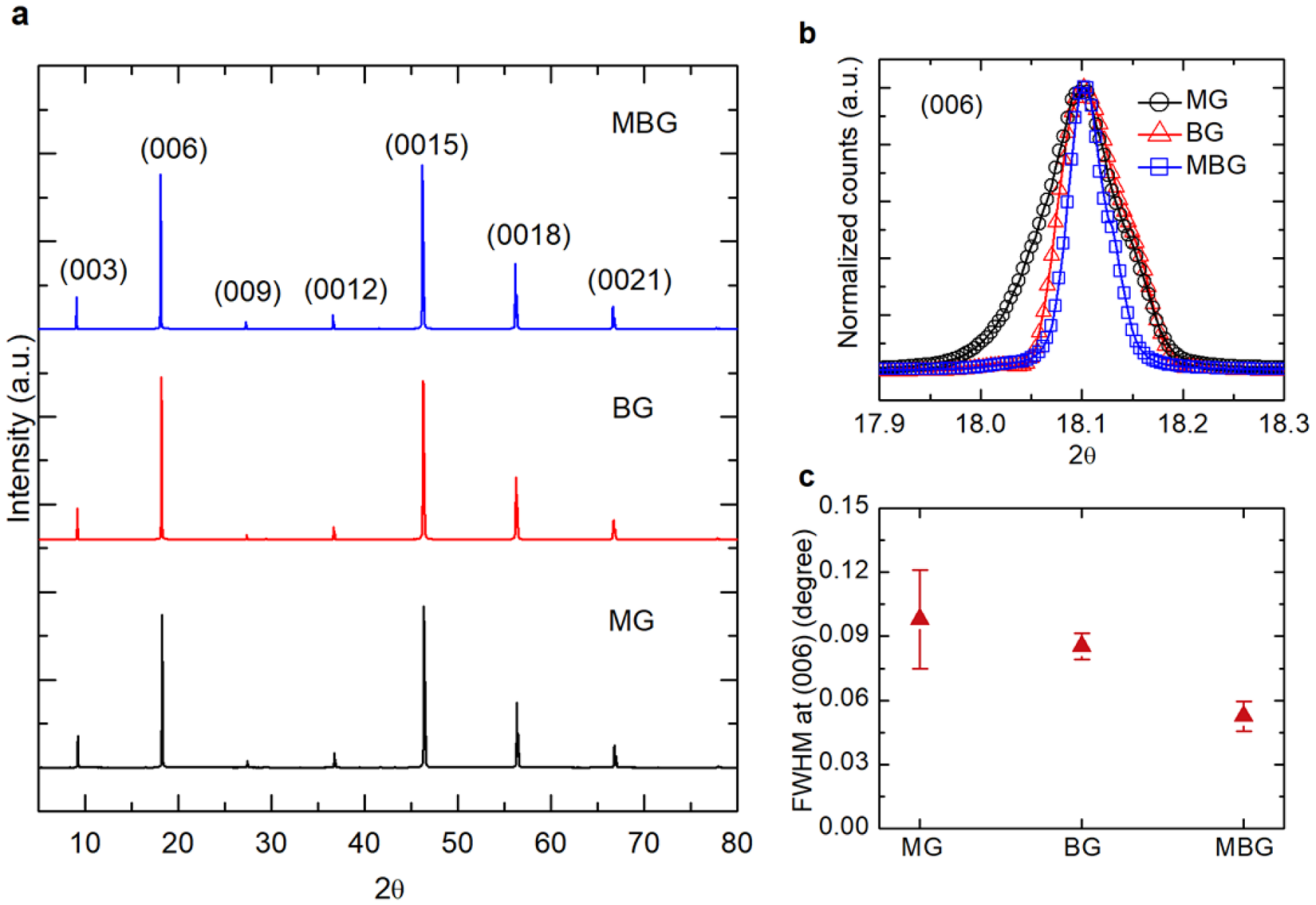
XRD patterns scanned at (00*l*) crystal surface for BSTS grown by MG, BG, and MBG methods (**a**). The (006) diffraction peaks (**b**) and their FWHMs (**c**) for the three BSTS growth methods. The FWHM are the mean value of the fitting of (006) diffraction peak from three representative XRD patterns for each sample. The error bars in (**c**) present the standard deviation of FWHMs of the (006) peak from three samples for each growth.

The crystal structure and crystal quality of the BSTS samples were studied by X-ray diffraction (XRD). Figure 2(a) shows the XRD patterns collected from the (00*l*) crystal surface. The BSTS thin flakes were cleaved from the center part of the crystal using a razor blade. The crystallographic planes of the corresponding diffraction peaks were well-indexed in the figure with no evidence of polycrystalline grains. The highest intensity diffraction peak, (006), for the BSTS samples grown by different methods is compared in Fig. 2(b). The MG BSTS showed a broad diffraction peak with two shoulders at both sides of the peak. The shoulder broadening could be due to the inhomogeneous compositions resulting in anti-site or vacancy defects in the crystals^14,18^. The diffraction peak of BG BSTS revealed only high angle broadening, suggesting a more homogeneous composition due to the zone melting and solidification process in the Bridgman method. Similar observations had been found for the vertical Bridgman growth in Bi_2_(Te_1−*x*_Se_*x*_)_3_ system^14^. The MBG BSTS showed a more symmetric Gaussian curve and narrower peak width, which indicated a better crystallinity and homogeneity compared to the MG and BG BSTS. For a quantitative comparison, the full-width at half maximums (FWHM) of the (006) diffraction peaks for the three BSTS growth methods are plotted in Fig. 2(c). The smaller FWHM generally implied the improvement in crystal lattice arrangement as observed in MBG BSTS.

### Crystal structure investigation

**Figure 3:**
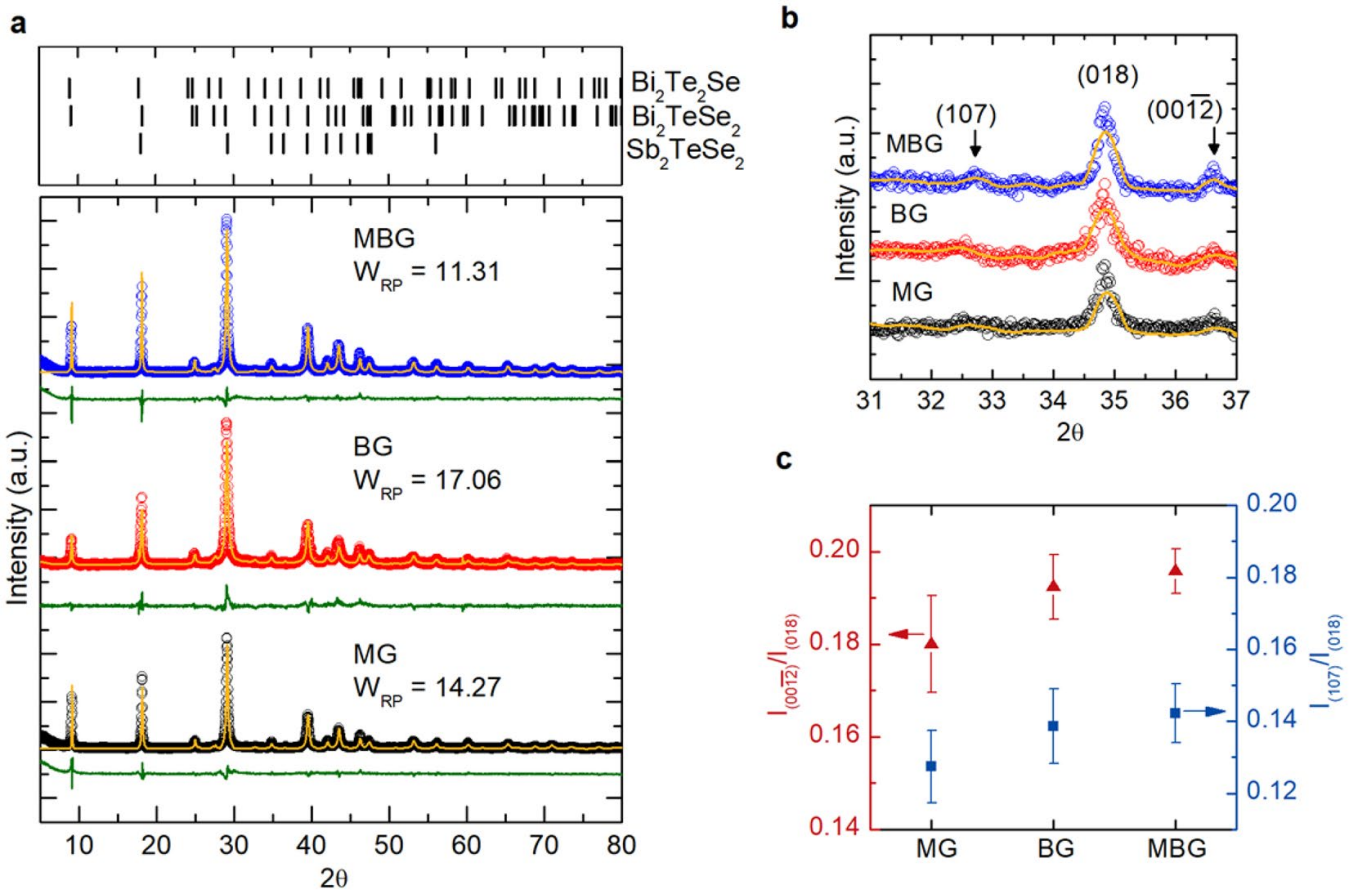
Powder XRD patterns of the BSTS samples grown by MG, BG and MBG methods (**a**). The extended view of the powder XRD patterns near the (108) peak to compare their characteristic diffraction peaks (**b**). The ${I}_{(00\overline{{\rm{12}}})}/{I}_{(018)}$ and ${I}_{(107)}/{I}_{(018)}$ of BSTS crystal grown by different methods (**c**). The solid dots and error bars in (**c**) present the mean values and standard deviation of the means calculated from three representative powder XRD patterns for each sample.

The crystal structures of the BSTS were examined from the powder XRD patterns as presented in Fig. 3(a). The upper panel presents the references for the Rietveld refinement (ICOD codes: 00-040-1211, 01-073-7366, and 01-089-2007). The solid dots and orange color lines are the measured and the refined powder XRD intensity. The green color lines show the difference between the measured and refined XRD. The Rietveld refinements from the diffraction data were well-matched to the rhombohedral structure in space group of ${\rm{R}}\bar{3}{\rm{m}}$. The weighted residual (W_RP_) indicates the residual of difference between observed and calculated intensities. The results agreed with the reported XRD data for BSTS topological insulators^18,20^.

Figure 3(b) compares the characteristic diffraction peaks of BSTS type chalcogenide^20^ located at 2θ ~32.5^o^ and 36.5^o^ for MG, BG and MBG BSTS. These peaks are corresponding to the (107) and $({\rm{00}}\overline{{\rm{12}}})$ crystal planes, respectively, where their appearances indicated the occupation of Se atoms in the center of the quintuple layer in our BSTS^20,27^. The peak intensities of their corresponding (107) and $({\rm{00}}\overline{{\rm{12}}})$ crystal planes have been used to characterize the Bi(Sb)/Te anti-site and Se vacancy defects in the BSTS crystal^13,17,20^. The higher intensities of (107) and $({\rm{00}}\overline{{\rm{12}}})$ diffraction peaks in MBG BSTS indicated the highly-ordered structure with relatively low composition-related defects compared to the MG and BG BSTS. The integrated peak intensity ratios of (107) and $({\rm{00}}\overline{{\rm{12}}})$ referred to (108), labeled as ${I}_{(00\overline{{\rm{12}}})}/{I}_{(018)}$ and ${I}_{(107)}/{I}_{(018)}$, respectively, for the three-types of BSTS samples are compared quantitatively and plotted in Fig. 3(c).

To further study the mosaicity of the BSTS crystal, we performed XRD in *ω* scan mode as shown in Fig. S2. Structure solution and refinement (as presented in Table S1) were performed with use of the SHELXTL (version 6.12) program package^28^. Face-indexed numerical absorption corrections were applied, since geometry of the crystal was 140 × 90 × 20 μm plate (Fig. S2(b)). From the Laue symmetry, the rhombohedral space group *R*$\bar{3}$*m* was deduced. The structure type, as expected was found to adopt Bi_2_Te_3_-type structure. Atomic mixing between Bi–Sb and Te–Se pairs was considered. Refined occupancies are within a reasonable agreement with the loaded composition, considering highly anisotropic geometry of the crystal, which greatly affects intensity data. Residual density is within typical values, considered heavy element composition. Mosaicity of the diffracted crystal is found to be 0.73°. Rocking curve plot for the *ω*-scan of 006 reflection shows FWHM to be 0.88° (Fig. S2(a)). The value of the FWHM for 006 reflection is used to compare crystallinity of the sample, and 0.88° result is comparable to non-epitaxially grown Bi_2_Te_3_ crystals^29^. The value of FWHM is rather low, considering high atomic disorder within the BiSbTeSe_2_ sample.

Furthermore, the atomic coordination, Wyckoff and site occupancy of the MBG BSTS obtained from the Rietveld refinement of the powder XRD pattern are analyzed in Table S2. BSTS has the tetradymite structure unit_,_ consisting of the stacked Te(Se)^1^-Bi(Sb)^1^-Te(Se)^2^-Bi(Sb)^1^-Te(Se)^1^. Since Se atom has more electronegativity than Te atom, the Se atom tends to occupy the central layer in BSTS structure unit^20^. The almost unity of Se^2^ occupancy (and much smaller Te^2^ occupancy) in Table S2 clearly elucidates this effect.

### Electrical transport

**Figure 4:**
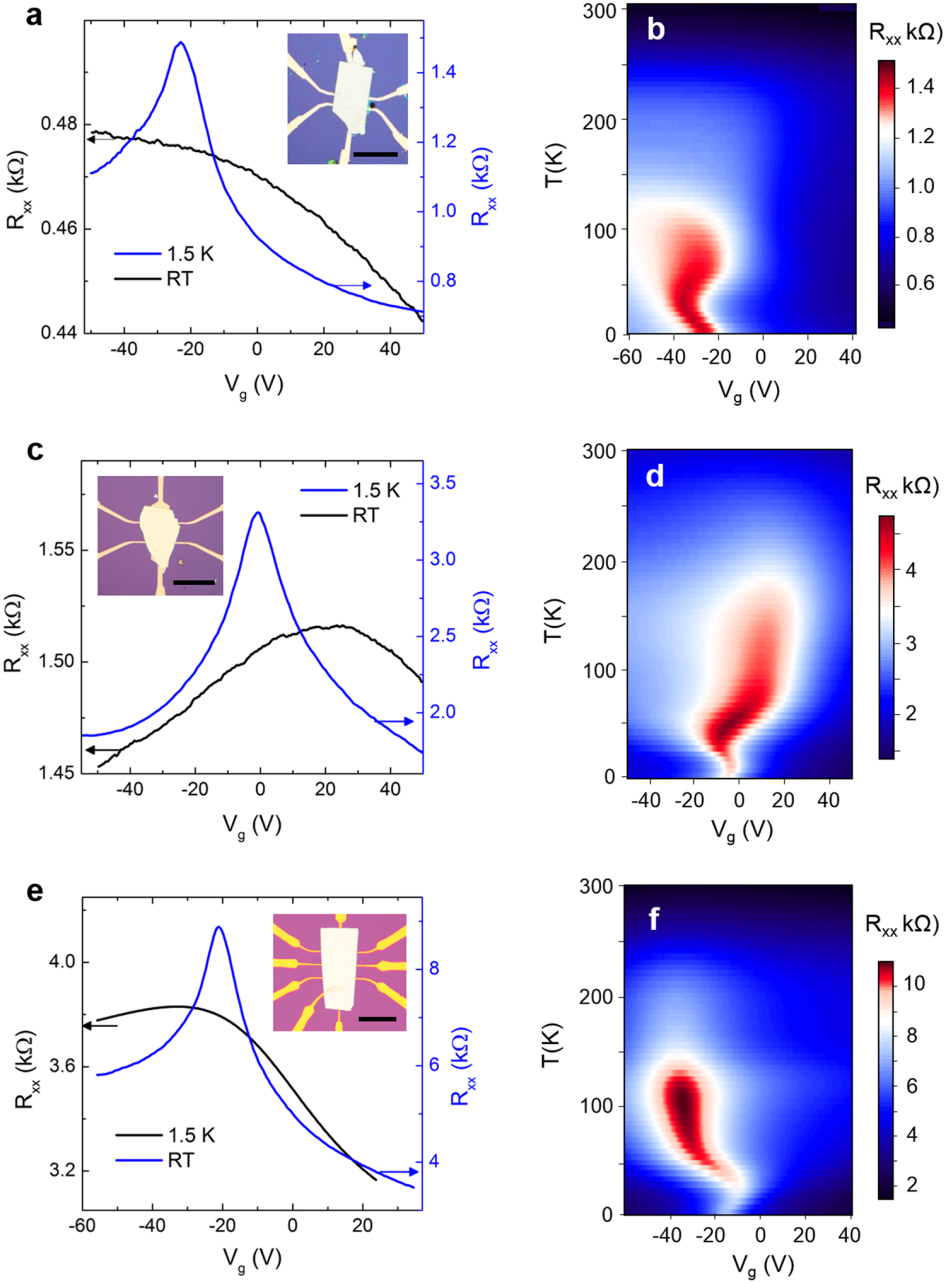
Gate-dependent resistances of MG (**a**), BG (**c**), and MBG (**e**) grown BSTS measured at RT and base temperature of 1.5 K. Optical images of the corresponding BSTS devices are inserted in (**a**,**c**) (scale bar = 50 μm). 2D color plots of the four-probe resistance as a function of temperature and gate voltage for MG (**b**), BG (**d**), and MBG (**f**) grown BSTS samples.

The electrical transport properties of the BSTS were studied to compare the quality of the crystals grown by different methods. Insets in Fig. 4(a,c,e) show the typical BSTS devices made by mechanical exfoliation and transfer onto pre-patterned electrodes on a Si/SiO_2_ substrate. The gate-dependent four-probe resistances of the BSTS measured at room temperature (RT) and 1.5 K are compared for the MG, BG, and MBG BSTS samples. All three BSTS samples display a broad resistance change over the gate voltage (V_g_) range at RT. The MG BSTS shows a larger change at positive V_g_ and nearly constant at negative V_g_; while the BG and MBG BSTS reveals a clear ambipolar signature at RT. The broad resistance peaks indicate a combination of bulk and surface conductions. The bulk contribution is more significant in the hole-conduction (constant R_xx_ region in negative V_g_) in MG BSTS. As the samples cooled down to base temperature (1.5 K), a sharp resistance peak was revealed in all samples. The distinct resistance peak is attributed to the surface transport due to the Dirac dispersion nature of the topological surface states as evidenced by angle-resolved photoemission spectroscopy in the stoichiometric BiSbTeSe_2_ compound^23^.

To further investigate the temperature dependent transport, the color maps of the R_xx_ as a function of temperature (y-axis) and gate-voltage (x-axis) for MG, BG, and MBG BSTS are shown in Fig. 4(b,d,f), respectively. For MG BSTS, the R_xx_ increased as the temperature decreases, and the resistance peak maximized at ~30 K. This insulating behavior is attributed to the bulk dominating conduction due to its insulating nature (bulk gap). The insulating trend terminated at about 30 K, suggesting the suppression of the BSTS bulk conduction at the temperature^25^. A gradual decrease in R_xx_ was observed by further reducing the temperature. As the surface states are gapless, this metallic behavior indicates prominently surface contribution to transport in this temperature range^25,30^. A similar temperature dependence profile was observed for the BG and MBG BSTS with resistance peak reaches maximum at ~50 K and ~100 K, respectively. To provide quantitative analyses on the bulk and surface conduction, we fit the four-probe resistivity (ρ_xx_) as a function of temperature for all three devices using a parallel conductance model^23^ as ${{\rm{\rho }}}_{{\rm{xx}}}=\frac{1}{{{\rm{G}}}_{{\rm{tot}}}}=\frac{1}{{{\rm{G}}}_{{\rm{bulk}}}+{{\rm{G}}}_{{\rm{surf}}}}$, where G_tot_, G_bulk_, and G_surf_ are the total, bulk and surface conductance, as presented in Fig. S3(a). Figure S2(b) shows that the G_bulk_ is negligibly small and the G_surf_ is dominating the transport at temperature below ~100 K for all the BSTS samples. Additionally, all three devices showed a visible shift in resistance peak position with temperature near the region when the R_xx_ reaching its maximum. This implied a shift of the chemical potential happened as the bulk conduction was suppressed and the total conduction developed into the surface conduction^30,31^. The origin of the resistance peak shifts is beyond the scope of the paper.

### Surface mobility study

**Figure 5:**
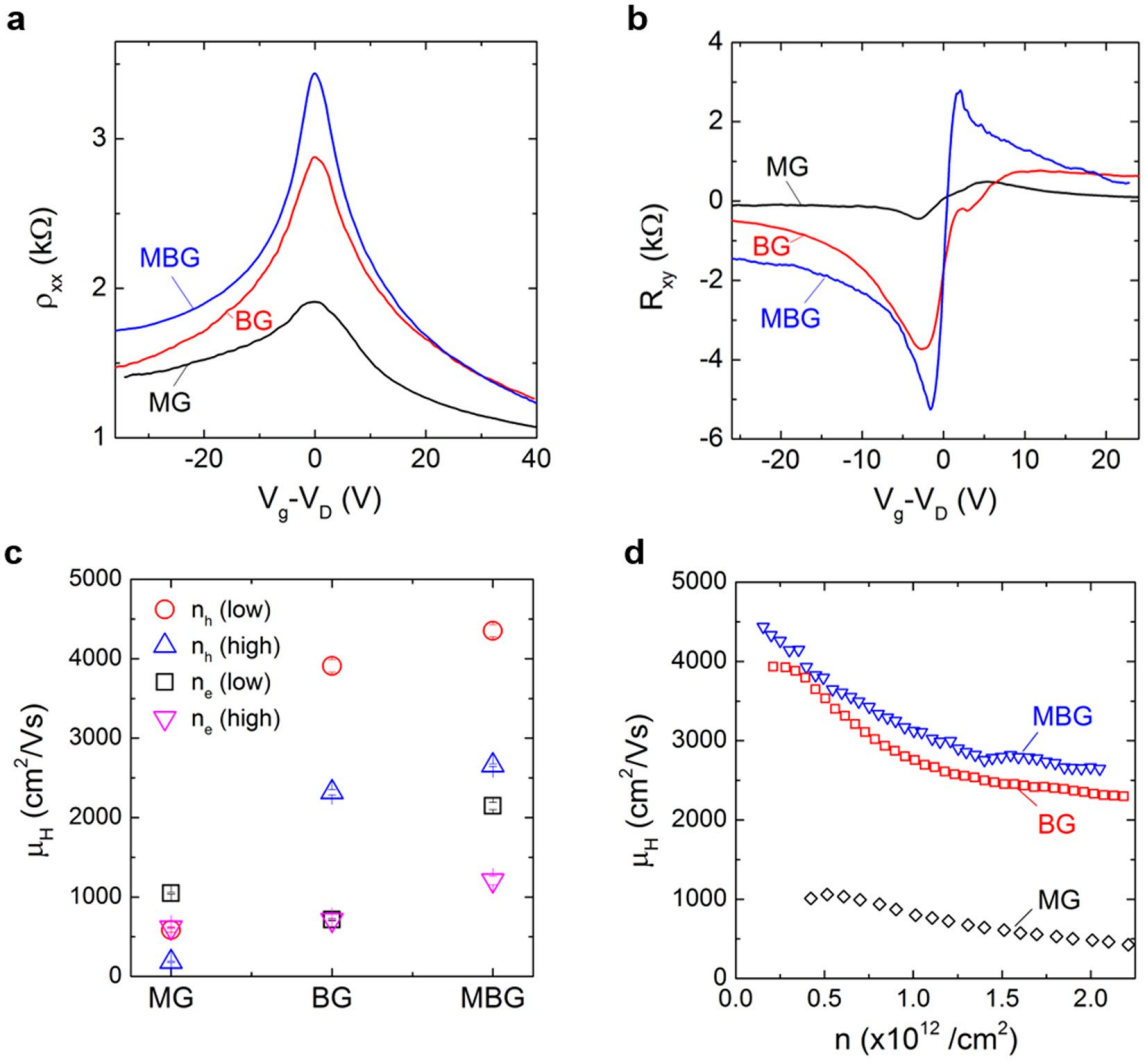
Longitudinal resistivity (**a**) and Hall resistance (**b**) of the MG, BG and MBG BSTS devices as a function of gate voltage measured at magnetic field of 0 T (**a**) and 2 T (**b**). Comparison of the Hall mobilities of the three-methods grown BSTS devices at different carrier densities at base temperature of 1.6 K (**c**). The error bars in (**c**) present the standard deviation of the Hall mobilities calculated at ±10% of the carrier densities (2 × 10^11^ cm^−2^ and 2 × 10^12^ cm^−2^ for low and high densities, respectively). Hall mobilities as a function surface charge density for BSTS grown by three different methods (**d**).

The low temperature magnetoelectric transport properties of three types of BSTS samples, grown by MG, BG, and MBG methods, are compared in Fig. 5. Figure 5(a) shows the longitudinal resistivity (ρ_xx_) of the MG, BG, and MBG BSTS devices as a function of gate voltage measured at base temperature (1.5 K) and zero magnetic field. Both BG and MBG BSTS reveal sharper resistivity peak and greater change in ρ_xx_ compared to the MG BSTS. This indicates a better quality of the BSTS grown by vertical Bridgman furnace. The resistivity peak width of the MBG is about 33% narrower than the BG BSTS. This is consistent with the observation of the narrower peak width from the XRD analyses. The Hall resistance (R_xy_) plots of the BSTS devices as a function of gate voltage measured at 2 T are compared in Fig. 5(b). The sign change in R_xy_ at the Dirac point confirms the ambipolar charge transport for all the BSTS devices. The R_xy_ of MBG BSTS (~2.7 kΩ/T) and BG BSTS (~2.0 kΩ/T) develop much faster than MG BSTS (~0.3 kΩ/T) in magnetic field. The R_xy_ of BG BSTS develops slower in the electron conduction region, while the MBG BSTS shows higher symmetry in both hole and electron conduction regions.

Hall mobility of the BSTS devices was calculated using the relation as: ${\mu }_{{\rm{H}}}=\frac{{{\rm{\rho }}}_{{\rm{xy}}}}{{{\rm{\rho }}}_{{\rm{xx}}}}\frac{1}{{\rm{B}}}$, from the linearly increasing region of R_xy_ versus B (0–2 T). Figure 5(c) compares the *μ*_H_ of the three BSTS devices at different charge density regions tuned by back-gate voltage. The *μ*_H_ of the MG BSTS maximized at ~1000 cm^2^/Vs in low electron density region. Both BG and MBG BSTS samples showed significantly higher *μ*_H_, which are about 3800 and 4400 cm^2^/Vs, respectively, in low hole density region. The mobility of our BG BSTS is comparable to values reported in literatures^23,25^ (see Fig. S5 for the comparison of the surface mobility in literatures). However, the *μ*_H_ in the electron region of BG BSTS is about five times smaller than the hole conduction region. The electron mobility of the BG BSTS is markedly improved by recrystallizing the single crystal, as revealed by MBG BSTS. The extremely high surface mobility in MBG BSTS is attributed to the enhanced crystallinity of its parent crystal as identified by XRD.

### Quantum transport

**Figure 6:**
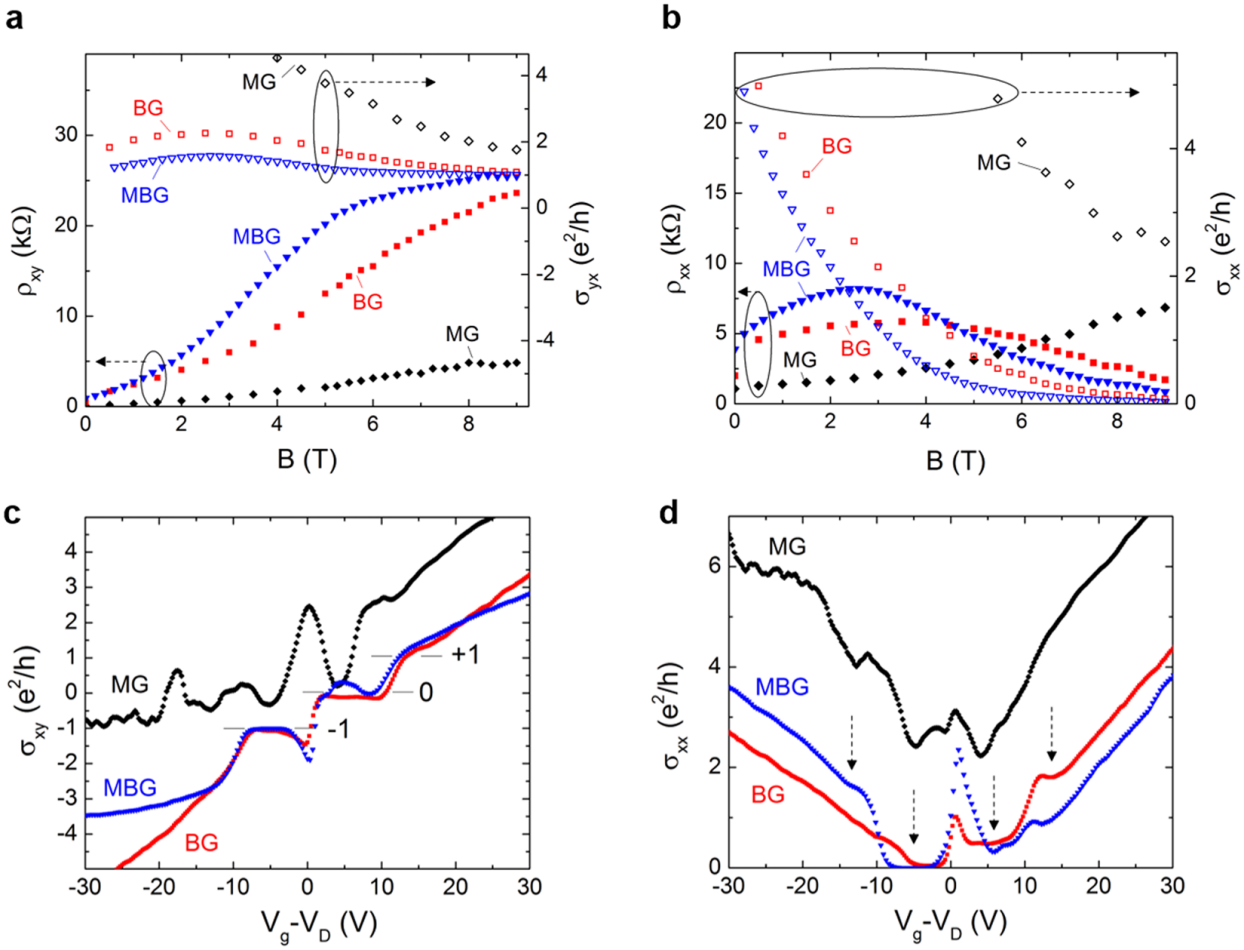
Plots of the Hall resistivity and conductivity (**a**), and the longitudinal resistivity and conductivity (**b**) as a function of magnetic field for the MG, BG and MBG BSTS devices. We noted that the MG BSTS is plotted in ρ_yx_ and σ_xy_ as its electron mobility is higher than the hole mobility. The Hall conductivity (**c**) and longitudinal conductivity (**d**) as a function of gate voltage at 9 T and 1.5 K for three methods grown BSTS devices.

The quantum magneto-transport of the three BSTS devices are compared in Fig. 6. The Hall resistivity (ρ_xy_), conductivity (σ_yx_) and longitudinal resistivity (ρ_xx_), conductivity (σ_xx_) of the MG, BG and MBG BSTS as a function of magnetic field are presented in Fig. 6(a,b), respectively. σ_xx_ and σ_yx_ are calculated from the relations as ${{\rm{\sigma }}}_{{\rm{xx}}}=\frac{{{\rm{\rho }}}_{{\rm{xx}}}}{({{\rm{\rho }}}_{{\rm{yx}}}^{2}+{{\rm{\rho }}}_{{\rm{xx}}}^{2})}$ and ${{\rm{\sigma }}}_{{\rm{yx}}}=\frac{{{\rm{\rho }}}_{{\rm{xy}}}}{({{\rm{\rho }}}_{{\rm{yx}}}^{2}+{{\rm{\rho }}}_{{\rm{xx}}}^{2})}$, respectively. For MG BSTS device, the ρ_xy_ and ρ_xx_ increase monotonically with magnetic field, which indicates that the surface states are in the normal Hall effect regime. For BG BSTS, the ρ_xy_ approaches the quantum limit (25.8 kΩ) of the integer quantum Hall effect (IQHE) at 9 T, together with the decreasing of ρ_xx_ at magnetic field above 4 T. Meanwhile, the ρ_xy_ of MBG BSTS reaches quantum Hall regime at magnetic field about 7 T as indicated by the saturation of ρ_xy_ and the suppression in ρ_xx_. The σ_yx_ are plateauing at +1e^2^/h at about 7 and 5 T for BG and MBG BSTS devices, respectively, as shown in Fig. 6(a). The corresponding σ_xx_ (Fig. 6(b)) vanishes to <0.1 e^2^/h. The development of IQHE in both BG and MBG BSTS suggests a better-quality BSTS crystal grown by Bridgman as compared to melting method.

To further confirm the IQHE in the BG and MBG BSTS, we investigate the quantization states by controlling the gate voltage. Figure 6(c,d) present the σ_xy_ and σ_xx_ of the MG, BG and MBG BSTS as a function of gate voltage. The MG BSTS shows no sign of σ_xy_ plateau formation at the any integer and the σ_xx_ is far from vanishing (>2 e^2^/h). On the other hand, the BG BSTS displays clear Landau level (LL) formation in σ_xy_ with filling factor, ν = −1, 0 and +1 by tuning the gate near the Dirac point. The LL states show different minima in σ_xx_ as indicated by the arrows. The IQHE has been observed in BG BSTS by several groups^23–25^. The IQHE is explained as a combination of half-integer QHE arising from the top and bottom surface states. The LL filling factor (ν) can thus be expressed as, ${\rm{\nu }}={{\rm{\nu }}}_{{\rm{t}}}+{{\rm{\nu }}}_{{\rm{b}}}$, with ${{\rm{\nu }}}_{{\rm{t}}}=({{\rm{n}}}_{{\rm{t}}}+\frac{1}{2})$ and ${{\rm{\nu }}}_{{\rm{b}}}=({{\rm{n}}}_{{\rm{b}}}+\frac{1}{2})$, where n_t_ and n_b_ are the LL indices of the top and bottom surfaces, respectively. For the MBG BSTS with higher mobility, an additional bump at σ_xy_ ~ 0.25 e^2^/h was observed in ν = 0 quantum state. This could be a developing quantum state, which requires further studies to confirm its origin.

## Discussion

Based on the observations, we showed that growth method has a strong effect on the crystal quality of the BSTS. We attributed this to the difference in growth mechanisms between the MG and BG methods. The MG sample solidifies from numerous nucleation sites along the wall of the ampoule. Whereas BG crystal nucleates from the bottom tip of the ampoule and solidifies along the vertical direction from bottom to the top. The multiple growth sites in MG are more likely to form grain boundaries when joining to form a bigger crystal. Crystal defects such as anti-sites, vacancies and other crystal disorder were also be reduced by the BG and MBG method. This minimized the electron scattering due to the defect sites in transport, and therefore resulted in a nearly four times enhancement in surface mobility of the BSTS. The enhancement in surface mobility led to the manifestation of IQHE in BG and MBG BSTS, in contrast to the MG BSTS.

Despite its high hole mobility, the electron mobility of the BG BSTS is relatively low (referred to Fig. 5(c)). We ascribed this to the imperfect composition stoichiometry of the BG BSTS, as indicated by the EDS and ICP-MS results. The asymmetry in electron and hole mobility has also been observed in graphene device and ascribed to the scattering of charge impurities from dopants^32^. As the stoichiometric BSTS is found to be n-type doping^23^, the light p-type doping behavior of BG BSTS at RT down to ~100 K (referred to Fig. 4(c,d)) also indicates its off-stoichiometry. The dopants in BG BSTS can act as charged impurities to limit the electron mobility. To analyze this effect, we plotted the field-effect mobility as a function of temperature for three types of BSTS devices in Fig. S4. In contrast to the MBG BSTS, the electron mobility (*μ*_e_) and hole mobility (*μ*_h_) reveal a distinct temperature dependence for the BG BSTS. The *μ*_e_ of BG BSTS vaguely changes with temperature (in comparison to the significant increase in *μ*_h_ at low temperature), which indicates a compensation in *μ*_e_ by charge impurities scattering despite a reduction in electron-phonon scattering at low temperature. Nevertheless, understanding of the origin of the electron-hole conduction asymmetry in doped BSTS will require a systematic study which is beyond the scope of this work.

Tuning the composition of the four elements is challenging because the stoichiometry of BiSbTeSe_2_ is not fixed thermodynamically (unlike Bi_2_Se_3_ or Bi_2_Te_2_Se) in phase diagram^33^. We argued that the two-step MBG method can resolve the inhomogeneous composition by recrystallizing the MG BSTS single crystal. The recrystallization in vertical Bridgman rearranged the molecules into ordered structure by directionally melting and solidifying, as indicated by the XRD patterns of the MBG BSTS. In addition, the quartz rod added on top of the single crystal ingot (made of the MG BSTS) minimized the headspace of the ampoule. This essentially limited the space for the vapor molecules to circulate in the ampoule, and therefore led to a composition more closely matching the desired stoichiometry^26^.

## Methods

### Ampoule preparation

The metal trace of bismuth (Bi), antimony (Sb), tellurium (Te), and selenium (Se) (Sigma-Aldrich Co., purity 5N grade). The quartz tubes (Technical Glass Products, Inc.) with outer diameter of 1.4 cm and 0.1 cm thickness of wall were used for ampoule preparation. The raw materials were weighed to the molar ratio of 1:1:1:2 for Bi:Sb:Te:Se and mixed into a mixture with total weight of 5 g. The quartz tube was sealed one end into a conical shape. The inner wall of the tube was coated with an inert carbon layer via pyrolysis of acetone to prevent the reaction of the materials with the tube. The mixture in the quartz tube was flushed with argon gas a few times to displace the air out from the tube. This followed by an evacuation of the tube to a pressure of below 10^−6^ torr to ensure a high vacuum environment for the crystal growth. The tube was then sealed by a torch into an ampoule with length of 6–8 cm.

### Melting growth

Melting growth BSTS single crystals were prepared in a muffle furnace (F30438CM, Fisher Scientific, Waltham, MA). The ampoule was placed at the center of the muffle furnace. The temperature of the furnace was increased to 850 °C at a slow rate of 0.1 °C/min and was held for 48 h. A few times of intermittent mixing were performed by gently shaking the ampoule at the temperature. The ampoule was then cooled down to 550 °C at a rate of 0.1 °C/min, and was annealed at the temperature for 96 h. After that, the sample was slowly cooled down to room temperature at a rate of 0.1 °C/min. The temperature as a function of time for the melting growth method was illustrated in Fig. 1(a).

### Bridgman growth

Bridgman growth was carried out in a vertical Bridgman furnace with three coil heaters^34^. The ampoule was held by a thin string at one end and placed vertically at the level above first heating zone. While the other end of the string was attached to a motion controller. The temperatures were set to 670 °C, 770 °C and 500 °C for warm, hot and cold zones, respectively, with the sequence from top to bottom heaters. The temperature profile was illustrated in Fig. 1(b). The first heater acted as a pre-heater to prevent the deposition of Se vapors on the wall of the ampoule^35^. The ampoule was translated vertically downward through the furnace at a very slow rate of 0.6 cm/day.

### Two-step melting and Bridgman growth

The single crystal BSTS was first grown in the muffle furnace by following the steps discussed in melting growth process. The as-grown single crystal BSTS was placed in a new carbon coated quartz tube with a quartz rod placed on top of the sample. The second growth was carried out in the vertical Bridgman furnace in the same processes described in the Bridgman growth method.

### Samples characterization

The as-grown crystals were cleaved and exfoliated along the crystalline surface for energy-dispersive X-ray spectroscopy (EDS) characterization. EDAX EDS (equipped in FEI Quanta 600 field emission scanning electron microscopy), operating at an acceleration voltage at 15 kV, was utilized to obtain EDS signals. Elemental compositions of the crystal were also studied using an inductively coupled plasma mass-spectrometry (ICP-MS, Agilent 7500 series, Agilent Technologies Inc.). For specimen preparation, the BSTS flakes were dissolved into a mixed nitric acid (HNO_3_, 1.2 M) and hydrochloric acid (HCl, 0.3 M) solution. Bruker D2 Phaser X-ray diffractometer with Cu K_α_ radiation and a zero-background holder (G130706, MTI Co.) was employed for crystal plane and powder X-ray diffraction (XRD) data acquisition. The full-pattern Rietveld refinement was performed using GSAS-II software to determine the crystal structure. A small single crystal was picked from the surface of BiSbTeSe_2_ sample and examined on a Bruker PLATFORM single-crystal diffractometer equipped with a SMART APEX II CCD area detector with a graphite-monochromated radiation (Mo Kα, *λ* = 0.70296 Å). Intensity data were collected using *ω* scans at 7 different *ϕ* angles with a frame width of 0.3° and an exposure time of 15 s per frame.

### Transport measurements

The BSTS flakes were exfoliated by using scotch tape on a polydimethylsiloxane (PDMS, Sigma-Aldrich Co.) substrate and transferred onto the pre-patterned gold leads in a Hall bar configuration. Three representative devices fabricated from the BSTS crystals grown by MG, BG, and MBG methods were compared. The thickness of the MG, BG, and MBG BSTS devices are about 120 nm, 80 nm and 70 nm, respectively. The transport measurements were carried out at various temperature from room temperature down to 1.5 kelvin, and magnetic field up to 9 tesla. The Hall measurements were performed by a SR830 DSP lock-in amplifier, operating at frequency of 17.777 hertz and the constant AC excitation current of 100 nA. The DC gate voltage was applied to the gate electrodes by a Keithley 2400 source measure unit.

### Accession Codes

CCDC 1864200 contains the supplementary crystallographic data for this paper. These data can be obtained free of charge via www.ccdc.cam.ac.uk/data_request/cif, or by emailing data_request@ccdc.cam.ac.uk, or by contacting The Cambridge Crystallographic Data Centre, 12 Union Road, Cambridge CB2 1EZ, UK; fax: +44 1223 336033.

## Electronic supplementary material

Supplementary Information

## References

[1] Jamali M (2015). Giant spin pumping and inverse spin Hall effect in the presence of surface and bulk spin−orbit coupling of topological insulator Bi_2_Se_3_. Nano Lett..

[2] Kondou K (2016). Fermi-level-dependent charge-to-spin current conversion by Dirac surface states of topological insulators. Nat. Phys..

[3] Sarma SD, Freedman M, Nayak C (2015). Majorana zero modes and topological quantum computation. Npj Quantum Info..

[4] Kitaev AY (2003). Fault-tolerant quantum computation by anyons. Ann. Phys..

[5] Franz M (2010). Solid-state physics U-turns strictly prohibited. Nature.

[6] Hsieh D (2008). A topological Dirac insulator in a quantum spin Hall phase. Nature.

[7] Xia Y (2009). Observation of a large-gap topological-insulator class with a single Dirac cone on the surface. Nat. Phys..

[8] Zhang T (2009). Experimental demonstration of topological surface states protected by time-reversal symmetry. Phys. Rev. Lett..

[9] Qu DX (2010). Quantum oscillations and Hall anomaly of surface states in the topological insulator Bi_2_Te_3_. Science.

[10] Analytis JG (2010). Two-dimensional surface state in the quantum limit of a topological insulator. Nat. Phys..

[11] Brahlek M, Koirala N, Bansal N, Oh S (2015). Transport properties of topological insulators: Band bending, bulk metal-to-insulator transition, and weak anti-localization. Solid State Comm..

[12] Koirala N (2015). Record surface state mobility and quantum Hall effect in topological insulator thin films via interface engineering. Nano Lett..

[13] Mi J (2012). Phase separation and bulk p-n transition in single crystals of Bi_2_Te_2_Se topological insulator. Adv. Mater..

[14] Jia S (2011). Low-carrier-concentration crystals of the topological insulator Bi_2_Te_2_Se. Phys. Rev. B.

[15] Jia S (2012). Defects and high bulk resistivities in the Bi-rich tetradymite topological insulator Bi_2+x_Te_2−x_Se. Phys. Rev. B.

[16] Xiong J (2012). Quantum oscillations in a topological insulator Bi_2_Te_2_Se with large bulk resistivity (6 Ω cm). Physica E.

[17] Ren, Z., Taskin, A. A., Sasaki, S., Segawa, K. & Ando, Y. Large bulk resistivity and surface quantum oscillations in the topological insulator Bi_2_Te_2_Se. *Phys. Rev. B* **82**, 241306(R) (2010).

[18] Taskin AA (2011). Observation of Dirac holes and electrons in a topological Insulator. Phy. Rev. Lett..

[19] Arakane T (2012). Tunable Dirac cone in the topological insulator Bi_2−x_Sb_x_Te_3−y_Se_y_. Nat. Commun..

[20] Ren Z (2011). Optimizing Bi_2−x_Sb_x_Te_3−y_Se_y_ solid solutions to approach the intrinsic topological insulator regime. Phy. Rev. B.

[21] Lee J (2012). Gate-tuned differentiation of surface-conducting states in Bi_1.5_Sb_0.5_Te_1.7_Se_1.3_ topological-insulator thin crystals. Phys. Rev. B.

[22] Segawa K (2012). Ambipolar transport in bulk crystals of a topological insulator by gating with ionic liquid. Phy. Rev. B.

[23] Xu Y (2014). Observation of topological surface state quantum Hall effect in an intrinsic three-dimensional topological insulator. Nat. Phys..

[24] Zhang S (2017). Anomalous quantization trajectory and parity anomaly in Co cluster decorated BiSbTeSe_2_ nanodevices. Nat. Comm..

[25] Li C (2017). Interaction between counter-propagating quantum Hall edge channels in the 3D topological insulator BiSbTeSe_2_. Phys. Rev. B.

[26] Bhat, H. Introduction to crystal growth: principles and practice. (ed. Bhat, H.) (CRC Press, 2015).

[27] Nam, H. *et al*. Microscopic investigation of Bi_2−x_Sb_x_Te_3−y_Se_y_ systems: On the origin of a robust intrinsic topological insulator. *J. Phys. Chem. Solids* corrected proof. (2017)

[28] SHELXTL; Bruker AXS Inc.: Bruker AXS Inc.: Madison, WI (2001).

[29] Collins-McIntyre LJ (2015). Growth of Bi_2_Se_3_ and Bi_2_Te_3_ on amorphous fused silica by MBE. Phys. Status Solidi B.

[30] Xia B (2013). Indications of surface-dominated transport in single crystalline nanoflake devices of topological insulator Bi_1.5_Sb_0.5_Te_1.8_Se_1.2_. Phys. Rev. B.

[31] Kim D (2012). Intrinsic electron-phonon resistivity of Bi_2_Se_3_ in the topological regime. Phys. Rev. Lett..

[32] Srivastava PK, Arya S, Kumar S, Ghosh S (2017). Relativistic nature of carriers: Origin of electron-hole conduction asymmetry in monolayer graphene. Phys. Rev. B.

[33] Kushwala SK (2016). Sn-doped Bi_1.1_Sb_0.9_Te_2_S bulk crystal topological insulator with excellent properties. Nat. Comm..

[34] Nagaoka A, Yoshino K, Taniguchi H, Taniyama T, Miyake H (2011). Growth of Cu_2_ZnSnS_4_ Single Crystal by Traveling Heater Method. Japanese J. Appl. Phys..

[35] Miyake H, Sugiyama K (1993). Single crystal growth of Cu-III-VI_2_ semiconductos by THM. Japanese J. Appl. Phys..

