# Supplementary material for: Enhancement in surface mobility and quantum transport of Bi2−xSbxTe3−ySey topological insulator by controlling the crystal growth conditions
Source: Sci Rep. 2018 Nov 23;8:17290. doi: 10.1038/s41598-018-35674-z (PMC6251917; doi:10.1038/s41598-018-35674-z)
Supplement: Supplementary file 1 — Supplementary Information [file 41598_2018_35674_MOESM1_ESM.pdf]

Supplementary information

Enhancement in surface mobility and quantum transport of $Bi_{2-x}Sb_xTe_{3-y}Se_y$ topological insulator by controlling the crystal growth conditions

Kyu-Bum Han[1†], Su Kong Chong[2†], Anton O. Oliynyk[3], Akira Nagaoka[4], Suzanne Petryk[5], Michael A Scarpulla[1,4], Vikram V. Deshpande[2], and Taylor D. Sparks[1,*]

[1]Department of Materials Science and Engineering, University of Utah, Salt Lake City, Utah 84112 USA

[2]Department of Physics and Astronomy, University of Utah, Salt Lake City, Utah 84112 USA

[3]Department of Chemistry, University of Alberta, Edmonton, AB T6G 2G2 Canada

[4]Department of Electrical Engineering, University of Utah, Salt Lake City, Utah 84112 USA

[5]Department of Computer Science, Cornell University, 402 Gates Hall, Ithaca, NY 14853, USA

[†]These authors contributed equally.

*Corresponding author: sparks@eng.utah.edu

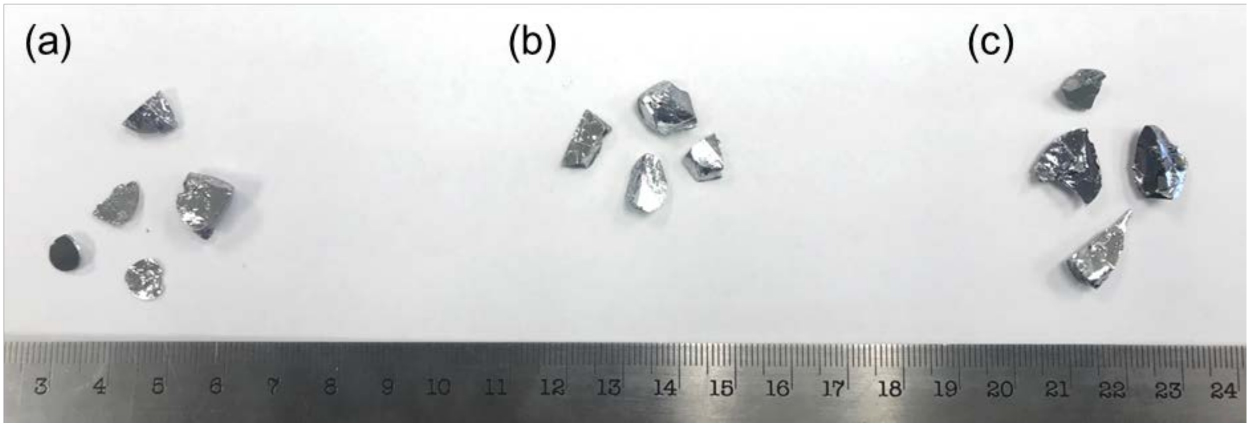

Figure S1. Photographs of the BSTS single crystals grown by melting (a), vertical Bridgman (b), and two-step melting & Bridgman (c) growth methods.

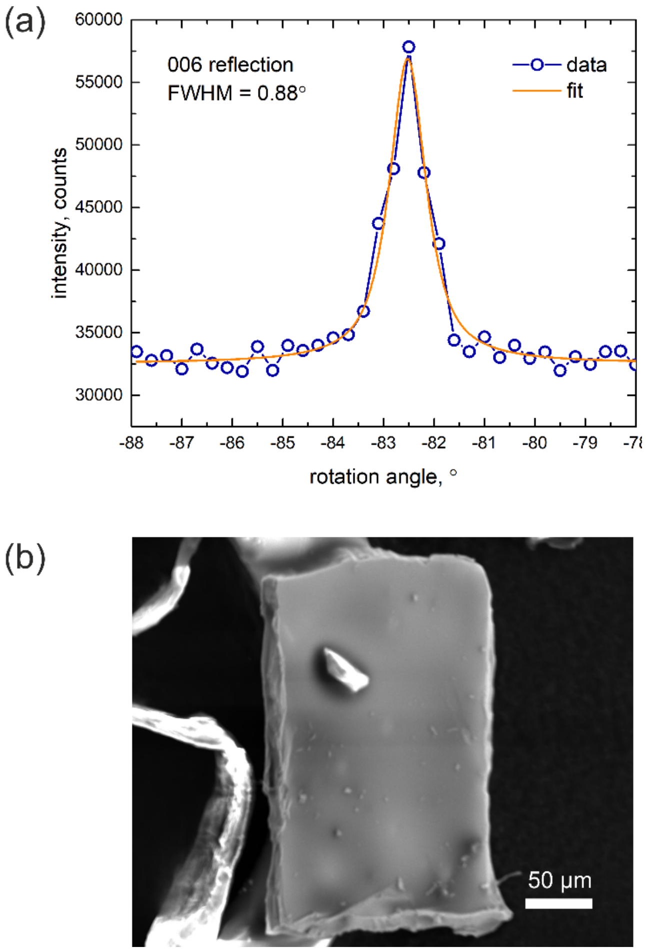

Figure S2. (a) rocking curve, ω-scan of 006 reflection; (b) SEM image of the single crystal BSTS flake.

Table S1. Crystallographic data for $BiSbTeSe_2$

Formula (refined occupancies)	$Bi_{1.12}Sb_{0.88}Te_{1.36}Se_{1.64}$
Formula mass (amu)	616.25
Space group	$R\bar{3}m$ (No. 166)
a (Å)	4.187(2)
c (Å)	29.648(16)
V (Å^3)	450.0(4)
Z	3
ρ_{calcd} (g cm^{-3})	6.822
T (K)	273(2)
Crystal dimensions (mm)	0.14 × 0.09 × 0.02
μ(Mo $K\alpha$) (mm^{-1})	50.530
Transmission factors	0.0553–0.4632
2θ limits	11.334–61.233°
Data collected	$-6 \le h \le 5$, $-6 \le k \le 6$, $-37 \le l \le 41$
No. of data collected	923
No. of unique data, including $F_o^2 < 0$	201(R_{int} = 0.0309)
No. of unique data, with $F_o^2 > 2\sigma(F_o^2)$	179
No. of variables	12
$R(F)$ for $F_o^2 > 2\sigma(F_o^2)$ [a]	0.0471
$R_w(F_o^2)$ [b]	0.1043
Goodness of fit	1.112
$(\Delta\rho)_{max}$, $(\Delta\rho)_{min}$ (e Å^{-3})	3.994, –1.816

[a] $R(F) = \sum||F_o| - |F_c|| / \sum|F_o|$. [b] $R_w(F_o^2) = [\sum[w(F_o^2 - F_c^2)^2] / \sum wF_o^4]^{1/2}$; $w^{-1} = [\sigma^2(F_o^2) + (Ap)^2 + Bp]$, where $p = [\max(F_o^2,0) + 2F_c^2] / 3$.

Table S2. Atomic coordination for $BiSbTeSe_2$

Atom	Occupancy	Wyckoff position	*x*	*y*	*z*	U_{eq} (Å^2) [a]	$U_{11} = U_{22}$ (Å^2)	U_{33} (Å^2)
Bi 1	0.56(6)	6*c*	0	0	0.39654(4)	0.0259(5)	0.0206(5)	0.0365(8)
Sb 1	0.44(6)	6*c*	0	0	0.39654(4)	0.0259(5)	0.0206(5)	0.0365(8)
Te 1	0.65(8)	6*c*	0	0	0.21296(7)	0.0284(6)	0.0228(6)	0.0395(12)
Se 1	0.35(8)	6*c*	0	0	0.21296(7)	0.0284(6)	0.0228(6)	0.0395(12)
Te 2	0.06(8)	3*a*	0	0	0	0.0194(9)	0.0170(10)	0.0241(17)
Se 2	0.94(8)	3*a*	0	0	0	0.0194(9)	0.0170(10)	0.0241(17)

[a] U_{eq} is defined as one-third of the trace of the orthogonalized U_{ij} tensor.

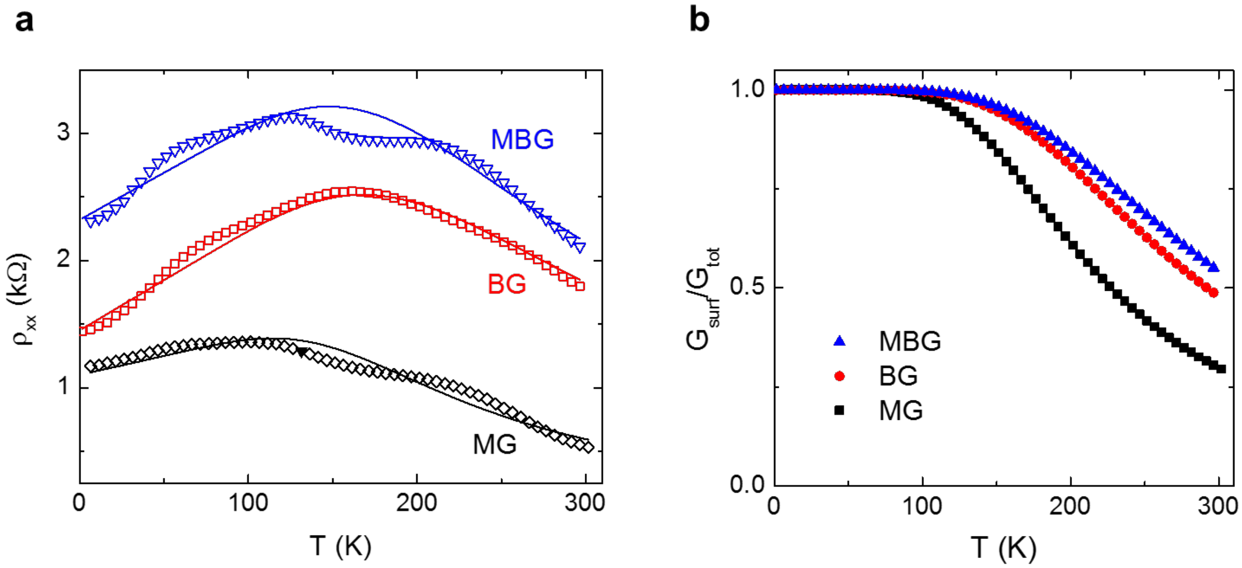

Figure S3. Plots of ρ_{xx} as a function of temperature (a) for the MG, BG, and MBG BSTS at gate-voltage away from Dirac point (high charge density regions). The hollow dots are the experimental data, and the solid lines present the fitting of ρ_{xx} from a parallel conductance model as described in the main text. The G_{bulk} and G_{surf} are calculated from the relations as: $G_{bulk}=\frac{t}{\rho_{3D}e^{\frac{\Delta}{k_B T}}}$ and $G_{surf}=\frac{1}{\rho_{2D}+AT}$, where t, Δ, A, ρ_{2D}, ρ_{3D}, and k_B are the thickness, activation energy, parameter for electron-phonon scattering, sheet resistance due to impurity scattering, bulk resistivity, and Boltzmann constant, respectively. The G_{surf} to G_{tot} ratio (G_{surf}/G_{tot}) as a function of temperature (b) for the MG, BG, and MBG BSTS.

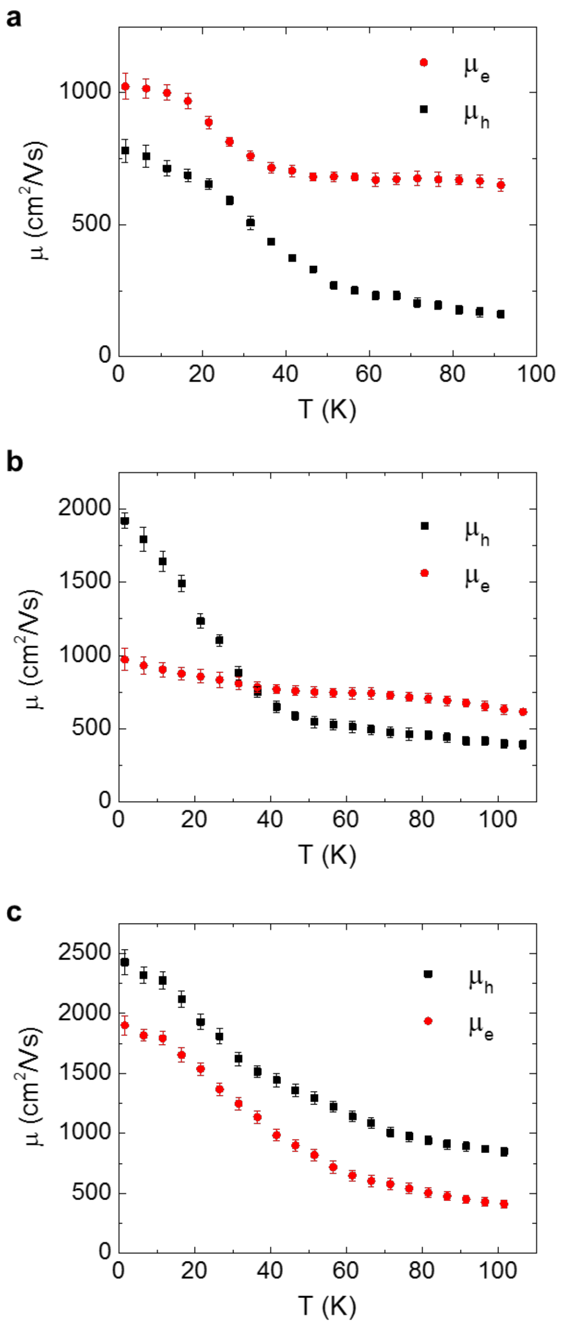

Figure S4. Plots of field effect mobility (μ) as a function temperature for (a) MG, (b) BG, and (c) MBG BSTS devices. The μ is calculated from the longitudinal resistiviy (ρ_{xx}) as $\mu = \frac{1}{C_g}\frac{\partial(\rho_{xx})^{-1}}{\partial V_g}$, where C_g is SiO_2 gate capacitance (thickness of SiO_2 ~300 nm and dielectric constant ~3.7). The temperature dependent μ are plotted up to 100 K as the surface conduction is dominating in the temperature range. The error bars are determined from the standard deviation of μ at ±10% of the carrier densities.

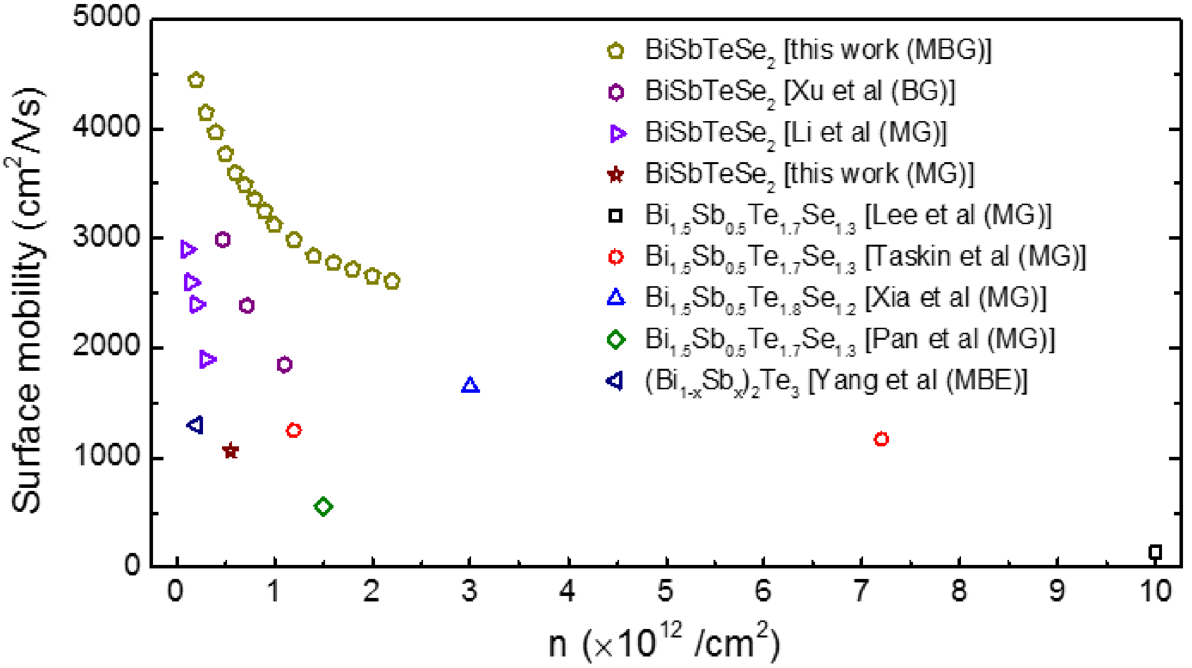

Figure S5. Comparison of the surface mobility with surface charge density of the single crystal $Bi_{2-x}Sb_xTe_{3-y}Se_y$ thin flake devices grown and measured in this works and by different groups using vertical Bridgman growth (BG)[1], melting growth (MG)[2-6], and molecular beam epitaxy (MBE)[7] methods. Only the literatures with the known density and mobility are compared in this plot.

References

1. Xu, Y. *et al.* Observation of topological surface state quantum Hall effect in an intrinsic three-dimensional topological insulator. *Nat. Phys.*, **10**, 956-963 (2014).
2. Li, C. *et al.* Interaction between counter-propagating quantum Hall edge channels in the 3D topological insulator $BiSbTeSe_2$. *Phys. Rev. B*, **96**, 195427 (2017).
3. Lee, J. *et al.* Gate-tuned differentiation of surface-conducting states in $Bi_{1.5}Sb_{0.5}Te_{1.7}Se_{1.3}$ topological-insulator thin crystals. *Phys. Rev. B*, **86**, 245321 (2012).
4. Taskin, A. A. *et al.* Observation of Dirac holes and electrons in a topological Insulator. *Phy. Rev. Lett.*, **107**, 016801 (2011).
5. Xia, B. *et al.* Indications of surface-dominated transport in single crystalline nanoflake devices of topological insulator $Bi_{1.5}Sb_{0.5}Te_{1.8}Se_{1.2}$. *Phys. Rev. B*, **87**, 085442 (2013).
6. Pan, Y. *et al.* Quantum oscillations of the topological surface states in low carrier concentration crystals of $Bi_{2-x}Sb_xTe_{3-y}Se_y$. *Solid State Comm.*, **227**, 13 (2016).
7. Yang, F. *et al.* Top gating of epitaxial $(Bi_{1-x}Sb_x)_2Te_3$ topological insulator thin films. *Appl. Phys. Lett.*, **104**, 161614 (2014).
